# Unveiling the growing significance of metabolism in modulating immune cell function: exploring mechanisms and implications; a review

**DOI:** 10.1097/MS9.0000000000001308

**Published:** 2023-09-13

**Authors:** Nicholas Aderinto, Muili Opeyemi Abdulbasit, Adrien Djabo Eric Tangmi, John Olalekan Okesanya, Jolayemi Mustapha Mubarak

**Affiliations:** a Department of Medicine and Surgery, Ladoke Akintola University of Technology, Ogbomoso; b Neuropsychiatric Hospital, Aro Abeokuta; c Faculty of Basic Medical Sciences, University of Ilorin, Ilorin, Nigeria; d Faculty of Medicine, Université Technologique Bel Campus, Kinshasa, DRC

**Keywords:** immunometabolism, metabolic dysregulation, metabolism

## Abstract

Immunometabolism has emerged as a rapidly growing field of research, holding significant promise for personalised medicine and precision immunotherapy. This review explores the intricate relationship between immune function and metabolic processes, emphasising their profound impact on various immune-related disorders. Understanding how metabolic dysregulation contributes to the pathogenesis of these disorders remains a critical research gap. Therefore, this review aims to bridge that gap by examining the key metabolic pathways involved and their specific implications in immune cell function. Key metabolic pathways, including glycolysis, mitochondrial metabolism, fatty acid metabolism, and amino acid metabolism, are discussed in the context of immune cell function. Dysregulation of these pathways can disrupt immune cell activation, differentiation, and overall function, contributing to disease pathogenesis. Understanding these metabolic alterations’ molecular mechanisms is essential for developing targeted therapeutic interventions. The review also emphasises the importance of personalised medicine in immune-related disorders. The unique metabolic profiles of individuals can influence treatment outcomes, highlighting the need for tailored approaches. Integrating metabolic profiling into clinical practice can enhance treatment efficacy and improve patient outcomes. Investigating the clinical significance of immunometabolism in diverse disease contexts will facilitate the translation of research findings into clinical practice. Moreover, refining treatment strategies based on individual metabolic profiles will contribute to advancing precision immunotherapy.

## Introduction

Highlights

- Metabolic processes play a crucial role in immune cell activation and govern their functionalities.
- Metabolic dysregulation has been implicated in immune-related disorders, offering potential avenues for therapeutic interventions.
- By targeting metabolic pathways, researchers aim to modulate immune responses and restore immune homoeostasis in various diseases.

The intricate relationship between intracellular metabolism and immunity has gained increasing attention recently. The burgeoning field of immunometabolism delves into the alterations in intracellular metabolism that accompany immune cell activation and govern their functionalities^1^. Metabolism plays a pivotal role in fuelling diverse biological processes, encompassing development, growth, differentiation, and the myriad functions executed by cells and tissues in fully matured adults^2^. Yet, cellular metabolism is not a fixed entity but rather an ever-changing phenomenon that dynamically adapts to cater to the bioenergetic demands of a cell at any given moment^3^. Upon activation, immune cells undergo substantial metabolic rewiring to meet the sudden surge in energy requisites and facilitate vital immune cell activities, including cytokine production, rapid cell proliferation, and motility^1^. The body mobilises the immune system in response to aberrant stimuli such as injuries, infections, and inflammation. In turn, immune cells respond to signals from neighbouring cells or alterations in their microenvironment by modulating their metabolic profiles^1^. This transition from a quiescent to an activated state entails a sequence of metabolic adaptations, predominantly involving converting intracellular energy sources and metabolic pathways^4^.

Recent advancements in immunology and metabolism have elucidated that intracellular metabolism exerts a notable influence on the differentiation and functionality of immune cells^5^. The interplay between metabolism and immune response, known as immune metabolism, is intricately entwined with immune activation in various disease contexts^5^. The activation, function, and differentiation of immune cells intrinsically rely on energy supply and metabolic alterations^6^. Furthermore, specific metabolic pathways within immunity have been linked to distinct immune functions, such as cytokine production. In contrast, immune molecules and pattern recognition receptors trigger diverse metabolic pathways within cells, regulated by oxygen levels^2^. Different immune cell subsets generate energy and biosynthetic intermediates using diverse metabolic routes. Despite their disparate end products, the metabolic pathways employed by immune cells exhibit interconnectedness, as they share common fuel inputs and rely on the products of one pathway to serve as synthetic precursors in alternative pathways^7^.

A comprehensive exploration of the role of intracellular metabolism in regulating immune cell function, both in health and disease, holds immense potential for unveiling novel therapeutic strategies with clinical applications. Our review aims to succinctly illuminate the intricacies of immune cell metabolism, regulatory mechanisms, the impact of metabolic disorders, potential therapeutic interventions, and future directions in immunometabolism.

## Methodology

This study aimed to investigate the emerging role of metabolism in modulating immune cell function by exploring the underlying mechanisms and discussing the implications. A search strategy was conducted across electronic databases, including PubMed, Scopus, and Web of Science to identify relevant articles. The search terms “metabolism,” “immune cell function,” “metabolic regulation,” “immune modulation,” “immune response,” and “immunometabolism” were used in various combinations. The search was limited to articles published between 2000 and March 2023. The inclusion criteria considered studies that examined the relationship between metabolism and immune cell function and provided mechanistic insights into how metabolic processes modulate immune cell function. They included studies conducted in vitro or in vivo using human or animal models. Articles written in English were included for better comprehension and accessibility. Conversely, exclusion criteria removed studies unrelated to the specific relationship between metabolism and immune cell function and non-English articles to avoid language barriers. These criteria aimed to select the study’s most relevant and reliable articles.

Following the removal of duplicate entries, the initial search yielded 500 articles. These articles underwent a thorough screening process based on evaluating titles and abstracts, resulting in the selection of 100 papers for full-text review. Subsequently, a rigorous analysis was conducted, and 94 articles that met the inclusion criteria were included in the final analysis. The selected articles underwent critical appraisal, and relevant data and information were systematically extracted. Key findings and concepts were identified, and a concise summary was constructed. It should be noted that a limitation of this study was the exclusion of non-English articles to maintain focus and consistency. This methodology ensured a meticulous and comprehensive approach to gathering relevant literature and examining the intricate relationship between intracellular metabolism and immunity within the field of immunometabolism.

### Overview of immune cell metabolism

**Figure 1:**
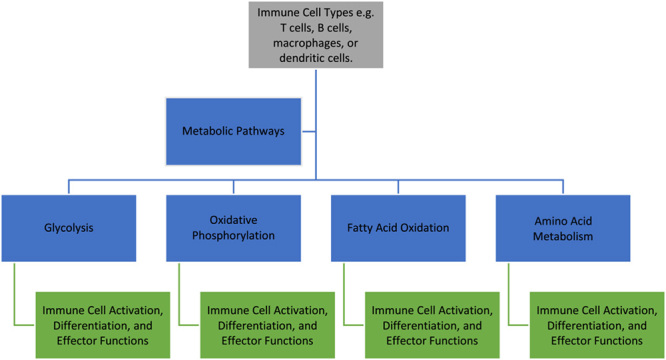
Overview of major metabolic pathways in immune cell function.

The orchestration of metabolic pathways plays a pivotal role in activating immune cells, furnishing the essential energy and molecular building blocks necessary for their effector functions^2^ (Figure 1). When confronted with stimuli such as infections or tissue injury, immune cells undergo a series of intricate metabolic reprogramming events, enabling them to meet the heightened energetic demands and support their activation^2^.

#### The glycolytic pathway

Glycolysis, an indispensable metabolic pathway initiated by cellular glucose uptake, holds paramount significance in immune cell metabolism^8^. While it produces a modest amount of adenosine triphosphate (ATP), glycolysis offers distinct advantages. It reduces nicotinamide adenine dinucleotide (NAD+) to NADH, serving as a vital cofactor for numerous enzymatic reactions^8^. Additionally, glycolysis allows the diversion of intermediate products to fuel biosynthetic growth pathways^8^. Cells often convert pyruvate to lactate to sustain glycolytic activity and recycle NADH^8^. Notably, glycolysis provides critical building blocks for nucleotide, amino acid, and fatty acid synthesis^8^.

Cell growth-associated signalling pathways actively promote the utilisation of glycolytic metabolism^9^. Consequently, glycolysis is dominant and indispensable in immune cell metabolism, serving as a pivotal energy source while supporting effector functions^9^. This metabolic pathway promotes cytokine production, facilitating immune cell activation and proliferation^10^. Immune cells rely heavily on glycolytic machinery to meet their heightened energy demands and execute their essential functions to mount an effective immune response^11^. Understanding the intricate interplay between glycolysis and immune cell metabolism provides valuable insights into the mechanisms governing immune cell activation and functioning. Further exploration of glycolytic regulation and its impact on immune responses may pave the way for novel therapeutic strategies in immunometabolism.

#### The tricarboxylic acid (TCA) cycle

The TCA cycle, widely recognised as the citric acid cycle, intricately orchestrates a closed loop of reactions, acting as a metabolic engine within cells^12^. This cycle receives diverse substrates and commences with the fusion of two-carbon acetyl-CoA and four-carbon oxaloacetate, culminating in the formation of citrate. Subsequent steps within the TCA cycle yield essential metabolic products, including NADH, carbon dioxide (CO_2_), ATP via guanosine triphosphate, and flavin adenine dinucleotide (FADH2)^12^. The cycle encompasses a sequence of conversions, progressing from citrate to isocitrate, α-ketoglutarate, succinyl-CoA, succinate, fumarate, malate, and ultimately regenerating oxaloacetate^12^. Notably, one of the enzymes involved in this cycle, succinate dehydrogenase, also functions as a component of the electron transport chain, thereby linking the TCA cycle to cellular respiration^13^.

The TCA cycle is fundamental to cellular metabolism by generating high-energy molecules, participating in biosynthetic processes, and maintaining redox balance^14^. The NADH and FADH2 produced during the TCA cycle serve as vital electron carriers, subsequently feeding into the electron transport chain to drive ATP synthesis through oxidative phosphorylation^14^. Additionally, the TCA cycle intermediates, such as α-ketoglutarate and succinyl-CoA, actively engage in diverse cellular pathways, including amino acid metabolism and lipid synthesis^14^. Integrating the TCA cycle with broader cellular metabolism is paramount in understanding the intricate balance and regulation of cell metabolic processes^14^. A comprehensive exploration of TCA cycle regulation and its impact on immune cell function holds immense potential for unravelling novel therapeutic avenues in immunometabolism^13^.

#### Cytosolic pentose phosphate pathway

The cytosolic pentose phosphate pathway, a vital metabolic route, fulfils diverse indispensable functions essential for cellular growth and survival^15^. This pathway is a critical diversion from glycolysis, redirecting intermediates towards generating nucleotide and amino acid precursors crucial for supporting cell proliferation through the non-oxidative branch^15^. Simultaneously, the oxidative branch of this pathway plays a pivotal role in generating NADPH, a key component in maintaining cellular redox balance and facilitating fatty acid synthesis^16^.

The non-oxidative branch of the cytosolic pentose phosphate pathway enables the efficient utilisation of intermediates derived from glycolysis, promoting the synthesis of nucleotide and amino acid building blocks necessary for cellular proliferation and growth^15^. By shunting these intermediates, the pathway supports essential biosynthetic processes that contribute to cell division and tissue regeneration. Concomitantly, the oxidative branch of the cytosolic pentose phosphate pathway plays a crucial role in redox homoeostasis and lipid metabolism^16^. By generating NADPH, this pathway provides a critical reducing power necessary to maintain the cellular redox balance and defend against oxidative stress^16^. Furthermore, NADPH is a vital cofactor in fatty acid synthesis, facilitating the production of lipids required for cellular membrane formation and energy storage^15^. The intricate interplay between the cytosolic pentose phosphate pathway, glycolysis, and cellular metabolism underscores its multifaceted significance in cellular physiology^16^.

#### Fatty acid oxidation pathway

Recent findings have shed light on the intricate relationship between metabolic pathways and the activation of the inflammasome in macrophages^17^. It has been revealed that in M1 macrophages, the inflammasome activation relies on fatty acid oxidation, while in M2 macrophages, glycolysis serves as the primary fuel for supporting fatty acid oxidation^17^. These novel insights highlight the potential of targeting lipid metabolism in macrophages to enhance therapeutic outcomes in metabolic diseases.

Fatty acid oxidation, a mitochondrial process, is crucial in converting fatty acids into energy-generating compounds such as acetyl-CoA, NADH, and FADH2^18^. The process begins with activating fatty acids in the cytosol, and then their transportation into the mitochondria^18^. Once inside the mitochondria, fatty acids undergo β-oxidation, producing acetyl-CoA, NADH, and FADH2^17^). These high-energy molecules are subsequently utilised in ATP production through the TCA cycle and the electron transport chain^18^.

The rate-limiting enzyme in fatty acid oxidation, carnitine palmitoyltransferase 1A (CPT1A), is crucial in regulating this metabolic pathway^19^. CPT1A is inhibited by malonyl-CoA, preventing excessive lipid oxidation during active lipid synthesis^19^. This mechanism ensures a fine-tuned balance between fatty acid oxidation and lipid synthesis, preserving cellular homoeostasis^18^. Importantly, fatty acid oxidation can yield substantial amounts of ATP, with a single palmitate molecule potentially producing over 100 ATP molecules^17^. This robust energy generation through fatty acid oxidation underscores its significance in supporting the energetically demanding functions of macrophages^19^.

#### Fatty acid synthesis

The fatty acid synthesis pathway is a fundamental process responsible for generating lipids critical for cellular growth and development^20^. It relies on precursor molecules derived from diverse metabolic pathways and is tightly regulated by signalling cascades, including the mechanistic target of rapamycin (mTOR) pathway. Key enzymes, such as sterol regulatory element-binding protein (SREBP), fatty acid synthase (FASN), and acetyl-CoA carboxylase (ACC), assume pivotal roles in orchestrating this intricate pathway^20^.

Fatty acid synthesis incorporates intermediate products derived from glycolysis, the TCA cycle, and the pentose phosphate pathway^21^. Acetyl-CoA, derived from citrate, is a crucial substrate that undergoes carboxylation, forming malonyl-CoA. The elongation of malonyl-CoA, catalysed by FASN, facilitates the generation of fatty acids. Notably, this process allows for the synthesis of fatty acids with varying chain lengths and unsaturated forms, including the production of branched-chain fatty acids^21^. These diverse fatty acids are essential building blocks for synthesising triacylglycerols and phospholipids, crucial components contributing to cellular structure and function^21^.

The regulation of the fatty acid synthesis pathway is tightly controlled to ensure proper lipid homoeostasis and cellular function. mTOR signalling significantly influences this pathway, integrating diverse intracellular and extracellular signals to modulate lipid synthesis^21^. SREBP, a transcription factor, acts as a central mediator of lipid metabolism, regulating the expression of key enzymes involved in fatty acid synthesis. FASN and ACC, among other enzymes, play vital roles in catalysing specific steps within the pathway, tightly controlling the overall lipid production process^21^.

#### Amino acid metabolic pathway

Amino acid metabolism is involved in various cellular processes. Amino acids serve as substrates for protein synthesis and are linked to important signalling pathways like mTOR and nucleotide synthesis. They can be utilised to synthesise branched-chain fatty acids^22^. Specific amino acids, such as glutamine and aspartate, are crucial for nucleotide synthesis. Glutamine can also be an alternative energy source and a precursor for fatty acid synthesis^23^. Other amino acids like arginine and tryptophan support cellular growth and proliferation through various metabolic pathways^22^.

Metabolic dysregulation, characterised by aberrant metabolic processes and disrupted energy homoeostasis, profoundly impacts immune cell function^2^. The intricate interplay between cellular metabolism and immune response is pivotal in orchestrating effective immune defence mechanisms. Consequently, disturbances in metabolic pathways can lead to dysfunctions in immune cell activation, differentiation, and effector functions, with far-reaching implications for overall immune response^23^.

Metabolic dysregulation affects many immune cells, including but not limited to macrophages, T cells, and B cells^24^. These cells undergo metabolic reprogramming in response to activation and encounter varying metabolic demands in different immune contexts. Glucose metabolism, specifically glycolysis, emerges as a central player in immune cell function, providing a rapid and robust energy source. Disruptions in glycolytic pathways can compromise immune cell responses, impairing cytokine production, cellular proliferation, and migration^25^.

Furthermore, altered lipid metabolism influences immune cell function and inflammation. Dysregulated fatty acid metabolism, such as imbalances in fatty acid synthesis and oxidation, can perturb immune cell signalling pathways and modulate inflammatory responses^26^. Lipid mediators derived from dysregulated lipid metabolism can impact immune cell activation, influencing the production of inflammatory cytokines and the resolution of immune responses^26^.

Mitochondrial dysfunction, another hallmark of metabolic dysregulation, profoundly affects immune cell metabolism and function^27^. Perturbed mitochondrial bioenergetics impair ATP production, leading to compromised immune cell activities and diminished immune responses. Additionally, mitochondrial reactive oxygen species generated during metabolic dysregulation can harm immune cells, impacting their functions and promoting oxidative stress^27^.

Understanding the impact of metabolic dysregulation on immune cell function holds significant clinical relevance. Metabolic disorders, such as obesity, diabetes, and metabolic syndrome, often coincide with compromised immune responses and increased susceptibility to infections and autoimmune diseases^28^. Insights into the intricate connections between metabolism and immune cell function may pave the way for novel therapeutic strategies targeting metabolic pathways to restore immune homoeostasis and improve clinical outcomes.

Traditionally, immune responses were predominantly attributed to molecular signalling pathways and immune cell interactions^29^. However, it has become increasingly evident that metabolic processes play a pivotal role in shaping immune cell fate and function^29^. Metabolism is responsible for providing energy and biosynthetic intermediates necessary for cellular activities. Importantly, metabolic processes are not merely passive bystanders but actively regulate immune cell functions and modulate immune responses^30^.

The interplay between metabolism and immune responses extends beyond energy production and nutrient utilisation^31^. Metabolic intermediates and signalling pathways directly impact immune cell signalling, gene expression, and epigenetic modifications^31^. Metabolic processes also contribute to immune cell fate decisions, determining whether a cell will differentiate into an effector or regulatory phenotype^6^. Dysregulation of immune metabolism has been implicated in various immune-related disorders, including autoimmune diseases, chronic inflammation, and cancer^6^. Moreover, immune metabolism is not limited to individual cells but extends to the complex interactions within the immune microenvironment^32^. Metabolic crosstalk between immune cells and other cell types, such as stromal cells, microbiota, and tumour cells, influences immune cell function, and disease outcomes^32^.

### Mechanisms of metabolic regulation of immune cell function

**Figure 2:**
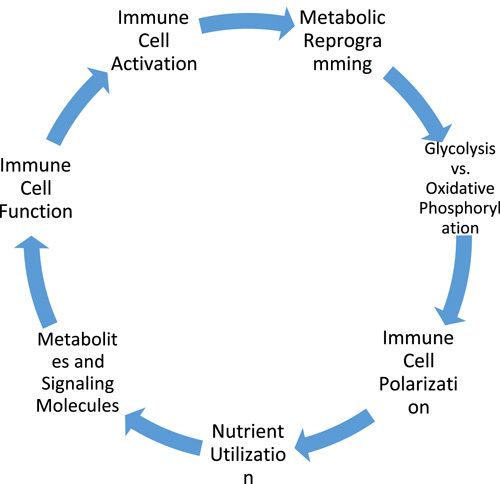
Influence of metabolic pathways on immune cell polarisation and function.

The immune system is a pivotal defense mechanism, diligently protecting the human body against the onslaught of pathogens and microorganisms that instigate infectious diseases^2^ (Figure 2). While immune cell interactions and signalling pathways are fundamental to its operation, a growing body of evidence highlights the significant contribution of metabolic processes, encompassing catabolism and anabolism, in shaping immune cell behaviour and functionality^33^.

#### Role of glucose metabolism in T-cell activation and differentiation

T cells, indispensable components of the adaptive immune system, orchestrate the defence against pathogens and uphold immune equilibrium^34^. The precise regulation of T-cell activation and differentiation, crucial for mounting effective immune responses, entails intricate interplay between various signalling pathways and metabolic processes^34^. Among these metabolic processes, glucose metabolism assumes a central role in shaping the fate and function of T cells.

Glucose, a vital cell energy source, is the primary fuel for T-cell activation and proliferation^35^. Upon encountering antigen-presenting cells, naïve T cells undergo rapid activation, triggering metabolic reprogramming to meet the heightened energy demands. This metabolic rewiring is characterised by augmented glucose uptake and glycolysis, channelling glucose towards the generation of biosynthetic precursors and sustaining T-cell effector functions^36^. Glycolysis emerges as a key driver of T-cell activation^34^. Activated T cells upregulate glucose transporters, including Glut1, facilitating increased glucose uptake and utilisation. This escalated glycolytic flux yields ATP and generates metabolic intermediates that fuel downstream biosynthetic pathways indispensable for T-cell growth and proliferation^37^. Moreover, glucose metabolism profoundly influences T-cell differentiation and effector function^36^. Distinct T-cell subsets, such as effector T cells (Teff) and regulatory T cells (Treg), exhibit disparate metabolic profiles that impact their respective functions^36^. Teff cells, actively engaged in pathogen clearance, exhibit elevated glycolytic rates to sustain robust effector responses. Conversely, Treg cells, critical for immune tolerance and regulation, rely on oxidative metabolism, encompassing fatty acid oxidation and mitochondrial respiration^38^.

Crucially, metabolic checkpoints, including the mTOR signalling pathway, tightly regulate glucose metabolism and T-cell fate determination. mTOR integrates signals from nutrient availability, growth factors, and cellular energy status to modulate T-cell metabolism and function^39^. Inhibition of mTOR complex 1 impedes glycolysis, compromising T-cell activation and effector function^40^.

The significance of glucose metabolism in T-cell activation and differentiation extends beyond energy production. Metabolites derived from glucose metabolism, such as acetyl-CoA and α-ketoglutarate, serve as pivotal substrates for epigenetic modifications and gene expression regulation^40^. These metabolic intermediates influence chromatin accessibility and transcriptional programs, ultimately moulding T-cell differentiation into distinct effector subsets^41^. The comprehensive understanding of the intricate interplay between glucose metabolism and T-cell biology holds therapeutic implications. Manipulating glucose metabolism presents novel avenues for modulating T-cell responses in diverse disease contexts. Targeting metabolic checkpoints, such as glucose transporters or enzymes involved in glycolysis, harbours potential for modulating T-cell activation and effector functions, offering prospects for autoimmune diseases, cancer immunotherapy, and vaccination strategies^42^.

#### Role of fatty acid metabolism in macrophage activation and polarisation

Macrophages, essential innate immune system components, play a pivotal role in host defence, tissue homoeostasis, and immune regulation^43^. The diverse functions of macrophages are intricately linked to their ability to adapt to various microenvironments and stimuli, resulting in distinct activation states and phenotypes^43^. Recent studies have shed light on the crucial role of fatty acid metabolism in macrophage activation and polarisation, influencing their immune functions and responses^44,45^.

Fatty acid metabolism encompasses the processes of fatty acid uptake, synthesis, and oxidation, which are critical for energy production and the maintenance of cellular functions^44^. The balance between fatty acid uptake, storage, and oxidation is tightly regulated and directly impacts macrophage activation and polarisation. There is a notable rewiring of fatty acid metabolism in pro-inflammatory or classically activated macrophages (M1)^44^. M1 macrophages exhibit increased fatty acid uptake and storage, accompanied by upregulation of fatty acid transporters, such as CD36. This augmented uptake of fatty acids supports the generation of lipid droplets, serving as intracellular reservoirs and sources of inflammatory lipid mediators, including eicosanoids and prostaglandins. Additionally, M1 macrophages elevate fatty acid oxidation (FAO) to generate ATP and maintain redox balance, facilitating robust pro-inflammatory responses^46^.

Conversely, alternatively activated or anti-inflammatory macrophages (M2) display distinct fatty acid metabolic profiles^46^. M2 macrophages prioritise fatty acid oxidation over storage, relying on FAO as a predominant energy source. This metabolic shift is associated with the upregulation of fatty acid transporters, such as fatty acid-binding protein 4, and enzymes involved in FAO, including carnitine palmitoyltransferase 1a (CPT1a)^47^. By favouring FAO, M2 macrophages generate ATP and produce metabolites contributing to anti-inflammatory and tissue-repairing functions^48^. Various signalling pathways and transcription factors further influence the dynamic interplay between fatty acid metabolism and macrophage activation. Notably, the nuclear receptor peroxisome proliferator-activated receptor gamma promotes an anti-inflammatory M2 phenotype by stimulating FAO and lipid uptake while inhibiting pro-inflammatory responses^48^. Conversely, toll-like receptor activation triggers pro-inflammatory M1 macrophage polarisation and enhances lipid storage through the activation of SREBPs and downstream lipogenic enzymes^49^.

Understanding the intricate relationship between fatty acid metabolism and macrophage activation holds therapeutic implications. Manipulating fatty acid metabolism presents a promising avenue for modulating macrophage functions in various diseases. Targeting key enzymes or transporters involved in fatty acid metabolism may provide opportunities to regulate macrophage activation, inflammation, and tissue repair in conditions such as atherosclerosis, obesity-related inflammation, and cancer^50^.

#### Role of amino acid metabolism in dendritic cell function

Dendritic cells are central in the immune system, acting as key orchestrators of immune responses and bridging the gap between innate and adaptive immunity^51^. Recent research has shed light on the pivotal role of amino acid metabolism in modulating dendritic cell function, influencing their antigen presentation capabilities and immune regulatory functions^51^.

Amino acid metabolism in dendritic cells encompasses a complex network of processes involved in the uptake, catabolism, and synthesis of amino acids. These are fundamental building blocks for protein synthesis and are indispensable for cellular functions^51^. Specific amino acids, including arginine, tryptophan, and glutamine, have emerged as critical players in dendritic cell biology and immune responses^51^.

The metabolism of arginine, predominantly regulated by the enzyme arginase, exerts profound effects on dendritic cell function^52^. By depleting arginine, arginase activity restricts the availability of this amino acid for nitric oxide synthesis and dampens T-cell activation. Consequently, arginase-expressing dendritic cells exhibit an immunosuppressive phenotype, fostering immune tolerance and the differentiation of regulatory T cells^51^. Another important metabolic pathway in dendritic cells involves tryptophan metabolism mediated by the enzyme indoleamine 2,3-dioxygenase (IDO). IDO catalyses the conversion of tryptophan into kynurenine, which exerts immunoregulatory effects^52^. Perturbations in tryptophan availability and the accumulation of kynurenine within the microenvironment impact dendritic cell maturation, T-cell activation, and immune tolerance. Enhanced IDO activity in dendritic cells has been associated with the induction of regulatory T cells and the suppression of effector T-cell responses, contributing to immune homoeostasis^52^.

Furthermore, glutamine metabolism plays a pivotal role in dendritic cell function by providing energy and biosynthetic intermediates^53^. Glutamine is a primary fuel source for dendritic cells, facilitating their activation, proliferation, and cytokine production. Moreover, glutamine metabolism influences dendritic cell maturation, antigen presentation, and T-cell activation. Modulating glutamine metabolism in dendritic cells holds the potential to impact immune responses and may offer therapeutic implications for diseases characterised by immune dysregulation^53^. Various signalling pathways and immune modulators govern the intricate interplay between amino acid metabolism and dendritic cell function. For instance, the mTOR pathway serves as a crucial regulator of amino acid sensing and metabolism, exerting profound influences on dendritic cell maturation and function^53^. Additionally, cytokines such as interferon-gamma, tumour necrosis factor-alpha, and transforming growth factor-beta can modulate amino acid metabolism in dendritic cells, shaping their immune regulatory functions^54^.

The elucidation of the impact of amino acid metabolism on dendritic cell function holds significant promise for therapeutic interventions. Manipulating amino acid metabolism pathways in dendritic cells may provide opportunities to modulate immune responses in various diseases, including cancer, autoimmune disorders, and immune-mediated pathologies.

The emerging understanding of the intricate interplay between immune cell function and metabolic processes holds immense potential for therapeutic innovations. Targeting metabolic pathways in immune cells opens avenues for precision medicines that restore immune homoeostasis and alleviate immune-related diseases. Innovative therapeutic strategies can optimise immune function and counteract immune-related disorders by manipulating metabolic checkpoints, modulating immune cell metabolic reprogramming, and exploiting metabolic vulnerabilities.

### The impact of metabolic dysregulation on immune-related disorders

Metabolic dysregulation profoundly impacts the development and course of immune-related disorders, unveiling a crucial interplay between metabolism and immune function. This intricate relationship is pivotal in autoimmune diseases, chronic inflammation, and immunodeficiency syndromes.

#### The role of metabolic disorders in autoimmune diseases

The role of metabolic disorders in the context of autoimmune diseases has garnered considerable attention due to emerging evidence suggesting their significant influence on disease pathogenesis, progression, and treatment outcomes^55^. Metabolic dysregulation, characterised by aberrations in glucose metabolism, lipid metabolism, and energy homoeostasis, can disrupt immune cell function, contribute to chronic inflammation, and play a role in developing autoimmune disorders^55^.

Glucose metabolism is a pivotal metabolic pathway implicated in autoimmune diseases. Abnormalities in glucose homoeostasis, such as insulin resistance and impaired glucose tolerance, have been observed in various autoimmune conditions, including type 1 diabetes, rheumatoid arthritis, and systemic lupus erythematosus^56^. Dysregulated glucose metabolism can impact immune cell activation, proliferation, and cytokine production, thus influencing immune responses and undermining immune tolerance mechanisms^55^. In addition to glucose metabolism, lipid metabolism has emerged as a critical player in autoimmune diseases. Lipids are important signalling molecules and structural components of cell membranes, exerting profound effects on immune cell function and inflammation^57^. Disrupted lipid metabolism, characterised by alterations in lipid profiles and increased adiposity, has been associated with several autoimmune conditions, such as multiple sclerosis and psoriasis^58^. Perturbed lipid metabolism can modulate immune cell activation, cytokine secretion, and the generation of lipid mediators, thereby exacerbating inflammatory responses and promoting autoimmune pathology.

Moreover, disturbances in energy homoeostasis have been implicated in autoimmune diseases. Obesity, a prevalent metabolic disorder characterised by excess adiposity, is closely intertwined with chronic low-grade inflammation and an elevated risk of developing autoimmune conditions like rheumatoid arthritis and systemic lupus erythematosus^59^. Adipose tissue, particularly visceral adipose tissue, acts as an active endocrine organ, secreting many adipokines, cytokines, and inflammatory mediators that can modulate immune cell function and contribute to immune dysregulation^60^.

The intricate interplay between metabolic disorders and autoimmune diseases involves complex mechanisms and pathways. Chronic inflammation, oxidative stress, alterations in gut microbiota, and dysregulated immune responses are among the key factors linking metabolic dysfunction to autoimmune pathogenesis^61^. Genetic predisposition and environmental factors also contribute to the development and progression of metabolic disorders and autoimmune diseases. Recognising the relationship between metabolic disorders and autoimmune diseases has substantial implications for patient management and therapeutic strategies. Targeting metabolic pathways and addressing metabolic abnormalities through lifestyle modifications, dietary interventions, and pharmacological approaches hold promise in ameliorating autoimmune disease outcomes^61^. Furthermore, active research areas include identifying metabolic biomarkers and developing personalised treatment regimens that consider both the immune and metabolic aspects of disease pathogenesis.

#### The role of metabolic disorders in cancer immunosuppression

The role of metabolic disorders in cancer immunosuppression is paramount, as it significantly influences the interplay between metabolism and immune function within the tumour microenvironment^62^. This intricate relationship shapes the tumour’s immune landscape and contributes to the evasion of immune surveillance, compromised anti-tumour immune responses, and resistance to immunotherapies.

Metabolic disorders, including obesity, diabetes, and dyslipidemia, induce systemic inflammation and disrupt cellular metabolism. These conditions foster an immunosuppressive milieu within the tumour microenvironment characterised by the accumulation of immunosuppressive cells such as myeloid-derived suppressor cells and regulatory T cells (Tregs), as well as the impairment of effector immune cell function. Obesity, marked by dysfunctional adipose tissue, promotes chronic low-grade inflammation by releasing pro-inflammatory adipokines and cytokines^62^. These factors influence the activation and function of immune cells, including tumour-infiltrating lymphocytes and antigen-presenting cells. Consequently, the dysregulated adipose tissue microenvironment hampers anti-tumour immune responses and fosters tumour progression.

In diabetes, hyperglycaemia directly impairs immune cell function, resulting in reduced cytotoxicity and impaired activation of T cells^63^. Insulin resistance and increased insulin-like growth factor 1 signalling sustain tumour cell survival and proliferation while inhibiting anti-tumour immune responses^63^. Dyslipidemia, characterised by elevated circulating lipid levels such as cholesterol and triglycerides, profoundly influences immune cell function and creates an immunosuppressive tumour microenvironment. Lipid metabolites, including free fatty acids, modulate immune cell signalling pathways and promote the differentiation of immunosuppressive cells like myeloid-derived suppressor cells and Tregs^63^. Furthermore, dyslipidemia alters the lipid composition of cell membranes, disrupting immune cell trafficking and function.

Metabolic disorders also contribute to the metabolic reprogramming of tumour cells and immune cells within the tumour microenvironment. Tumour cells undergo metabolic rewiring, favouring increased glycolysis and fatty acid synthesis to fulfil their energy requirements and support rapid proliferation^63^. This metabolic reprogramming creates a nutrient-deprived and immunosuppressive microenvironment, impairing the function of immune effector cells and facilitating tumour immune evasion.

#### The role of metabolic disorders in infectious diseases

Metabolic disorders substantially influence the dynamics of infectious diseases, wherein their presence can disrupt immune responses, induce immunosuppression, and modulate disease outcomes. Obesity, diabetes mellitus, and dyslipidemia are among the metabolic disorders that garnered attention due to their profound impact on the host-pathogen interplay.

Obesity has emerged as a risk factor for various infectious diseases. Adipose tissue dysfunction produces pro-inflammatory adipokines and cytokines, which impede immune cell function and compromise the body’s ability to effectively defend against invading pathogens^64^. Moreover, obesity-related mechanical and metabolic alterations in the respiratory system increase susceptibility to respiratory infections, exacerbating disease severity. Hyperglycaemia adversely affects multiple immune system components, including neutrophils, macrophages, and lymphocytes, impairing their antimicrobial activities and compromising pathogen eradication^64^. Furthermore, the complications associated with diabetes, such as peripheral neuropathy and impaired wound healing, heighten the risk of secondary infections and hinder recovery.

Dyslipidemia, characterised by abnormal lipid profiles, contributes to the modulation of infectious diseases. Elevated levels of circulating lipids, such as cholesterol and triglycerides, have been linked to impaired immune cell function, including compromised phagocytosis and antigen presentation^64^. Dyslipidemia can also promote the formation of atherosclerotic plaques, serving as reservoirs for certain pathogens and fostering chronic infections with heightened disease severity. Metabolic disorders affect both innate and adaptive immune responses, instigating a state of immune dysregulation. The chronic low-grade inflammation accompanying metabolic disorders fosters an environment that perpetuates tissue damage and impedes the immune system’s ability to mount an appropriate response against pathogens^64^. Additionally, metabolic dysregulation can disrupt the composition of the gut microbiota, disturbing the delicate equilibrium between commensal and pathogenic bacteria, further influencing immune homoeostasis.

The impact of metabolic disorders on infectious diseases extends beyond initial susceptibility and disease severity, encompassing implications for antimicrobial therapy and vaccine efficacy. Altered immune responses and compromised host defence mechanisms in individuals with metabolic disorders can undermine the effectiveness of antimicrobial agents, leading to treatment failure and prolonged recovery. Moreover, impaired vaccine responses in the context of metabolic disorders can undermine the effectiveness of vaccination strategies, heightening the risk of vaccine-preventable infections.

### Therapeutic targeting of metabolic regulators for immune-related disorders

The therapeutic targeting of metabolic regulators has emerged as a promising strategy for managing immune-mediated disorders. The intricate interplay between immune responses and cellular metabolism has revealed numerous potential targets for intervention to restore immune homoeostasis and mitigate disease pathology.

#### Potential therapeutic interventions targeting metabolic regulators for autoimmune diseases

One potential therapeutic target is the mTOR (mammalian target of rapamycin) signalling pathway, a central regulator of cellular metabolism and immune cell function^65^. Inhibitors of mTOR, such as rapamycin and its analogues, have shown promise in preclinical and clinical studies for treating autoimmune diseases^66,67^. By modulating T-cell activation and differentiation, these inhibitors exhibit immunosuppressive effects and hold the potential to attenuate autoimmune responses. Another promising target is the enzyme pyruvate kinase M2 (PKM2), which governs glycolysis in immune cells. Inhibition of PKM2 has demonstrated efficacy in preclinical models of autoimmune diseases by influencing immune cell metabolism and function^68^. By targeting PKM2, it may be possible to restore the equilibrium between effector and regulatory T cells, thereby exerting immunomodulatory effects.

Furthermore, manipulating key enzymes involved in amino acid metabolism, such as IDO and tryptophan 2,3-dioxygenase, holds potential therapeutic value. These enzymes play critical roles in immune tolerance and regulation. Inhibitors of IDO and tryptophan 2,3-dioxygenase have shown promising outcomes in preclinical studies, suggesting their potential as therapeutic agents for autoimmune diseases^69,70^. Additionally, the modulation of metabolic checkpoints involved in fatty acid metabolism, such as the enzyme ACC, presents an intriguing avenue for intervention. Inhibition of ACC can impact the balance of pro-inflammatory and anti-inflammatory lipid mediators, thereby influencing immune cell function and ameliorating autoimmune responses.

#### Potential therapeutic interventions targeting metabolic regulators for cancer immunotherapy

Cancer immunotherapy has emerged as a promising approach for treating various malignancies in recent years. However, many patients do not respond to immunotherapy or develop resistance over time. The complex interplay between tumour cells and the immune system, including metabolic dysregulation within the tumour microenvironment, plays a critical role in cancer progression and immunosuppression. Targeting metabolic regulators offers a potential strategy to overcome these challenges and enhance the efficacy of cancer immunotherapy.

One potential target is the enzyme IDO, which is upregulated in tumour cells and immune cells within the tumour microenvironment. IDO promotes immune tolerance by depleting tryptophan and generating immunosuppressive metabolites. Inhibitors of IDO, such as epacadostat, have shown promising results in preclinical studies and early clinical trials, demonstrating the ability to restore anti-tumour immune responses and improve patient outcomes^71^. In addition, a new metabolic regulator of interest is the adenosine monophosphate-activated protein kinase (AMPK). AMPK is a key regulator of cellular energy metabolism and is involved in maintaining cellular homoeostasis^72^. Activation of AMPK has been shown to enhance anti-tumour immune responses by promoting the infiltration and activation of immune cells in the tumour microenvironment^72^. Pharmacological activators of AMPK, such as metformin, are being investigated as potential adjuncts to cancer immunotherapy^72^.

The mTOR pathway is another metabolic regulator with implications for cancer immunotherapy. mTOR is a central regulator of cellular metabolism and growth^65^. Dysregulation of the mTOR pathway in tumours and immune cells can lead to immunosuppression. Inhibitors of mTOR, such as rapamycin and its analogues, have shown immunomodulatory effects and can enhance anti-tumour immune responses when combined with immunotherapeutic agents.

Furthermore, immune checkpoint inhibitors targeting programmed cell death protein 1 (PD-1) and its ligand PD-L1 have revolutionised cancer treatment^72^. These inhibitors unleash the anti-tumour immune response by blocking inhibitory signalling pathways. However, metabolic alterations within the tumour microenvironment can limit the effectiveness of immune checkpoint inhibitors. Combining metabolic modulators with immune checkpoint inhibitors may overcome these limitations and improve treatment outcomes.

### Interaction between metabolism and immune signalling

**Figure 3:**
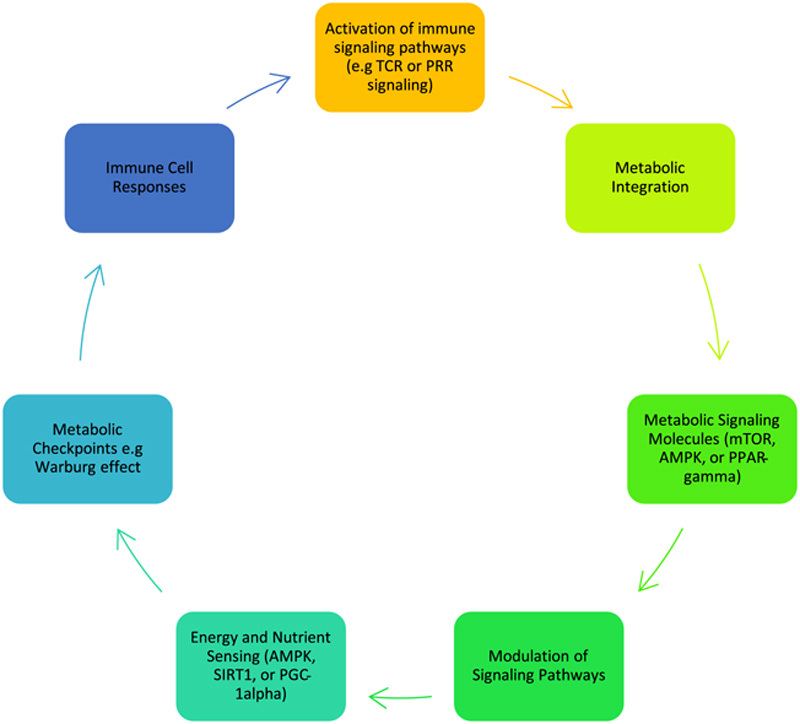
Interplay between metabolism and immune signalling. AMPK, adenosine monophosphate-activated protein kinase; mTOR, mechanistic target of rapamycin; PPAR, peroxisome proliferator-activated receptor; PGC,Peroxisome proliferator-activated receptor-gamma coactivator; PRR, pattern recognition receptor; SIRT1, Sirtuin 1; TCR, T Cell Receptor.

The reciprocal relationship between metabolism and immune signalling is a dynamic and intricate process that profoundly influences immune cell function^73^ (Figure 3). Immune signalling molecules, including cytokines and growth factors, play a pivotal role in modulating metabolic pathways in immune cells, while metabolic processes can, in turn, impact immune signalling^74^. Cytokines and growth factors, key immune signalling molecules, have been shown to exert regulatory control over metabolic pathways in immune cells. For instance, interleukin-6 has promoted glycolytic metabolism in T cells, thereby supporting their effector functions^75^. Similarly, interferon-gamma has been found to enhance FAO in macrophages, enhancing their inflammatory responses^76^. These examples highlight how immune signalling molecules can dynamically modulate metabolic processes to meet the specific functional requirements of immune cells. The mechanisms underlying immune signalling modulation of metabolic pathways are diverse. They can involve transcriptional regulation, post-translational modifications, and alterations in enzymatic activity. For instance, activating specific transcription factors, such as signal transducer and activator of transcription proteins, by immune signalling molecules can lead to the upregulation of metabolic genes involved in nutrient uptake, glycolysis, or mitochondrial function^77^. Furthermore, immune signalling can induce post-translational modifications of metabolic enzymes, altering their activity or subcellular localisation to promote metabolic adaptations in immune cells.

Conversely, metabolic processes can impact immune signalling in immune cells. The metabolic state of immune cells, influenced by nutrient availability and metabolic intermediates, can dictate immune cell activation, differentiation, and effector functions^78^. For example, increased glucose metabolism and glycolytic flux have been associated with enhanced T-cell activation and effector functions^17^. Metabolites derived from metabolic pathways, such as acetyl-CoA or alpha-ketoglutarate, can act as cofactors or substrates for epigenetic modifications and influence gene expression programs in immune signalling^79^. Moreover, mitochondrial function and metabolites derived from oxidative phosphorylation (OXPHOS) have emerged as critical regulators of immune cell signalling^80^. Mitochondrial ROS, generated during OXPHOS, can act as signalling molecules to modulate immune cell activation and inflammatory responses^81^. Furthermore, metabolites produced during OXPHOS, such as succinate or fumarate, can be intermediates in cellular signalling pathways, impacting immune cell function^14^.

Understanding the crosstalk between metabolism and immune signalling opens new avenues for developing innovative therapeutic strategies. Targeting both immune signalling and metabolic pathways holds immense promise for immunotherapy and the management of immune-related disorders. Modulating metabolic processes in immune cells may enhance immune responses against pathogens or tumours or, conversely, dampen hyperactive immune reactions in autoimmune diseases. For example, pharmacological interventions that target specific metabolic pathways or metabolic enzymes could be designed to regulate immune cell function. Manipulating the activity of metabolic regulators or metabolic sensors, such as AMPK or mTOR, could offer opportunities for immune modulation.

### Emerging trends and future perspectives in immunometabolism

The emerging field of immunometabolism has provided valuable insights into the intricate relationship between cellular metabolism and immune responses. These insights have significant implications for personalised medicine and the development of precision immunotherapy approaches. By understanding the metabolic profiles of individual patients and tailoring interventions to modulate immune cell metabolism, personalised medicine can revolutionise the treatment of immune-related disorders.

One potential implication is the identification of metabolic biomarkers that can serve as indicators of disease progression and treatment response. Metabolic profiling of immune cells and their microenvironment can provide valuable information about the metabolic alterations associated with specific diseases. These metabolic biomarkers can aid in early disease detection, prognostic assessment, and monitoring of treatment efficacy. By incorporating metabolic biomarkers into diagnostic and therapeutic algorithms, clinicians can make informed decisions and optimise treatment strategies for individual patients.

Immunometabolism also holds promise for guiding the selection and optimisation of immunotherapeutic interventions. The metabolic state of immune cells can influence their response to immunotherapies, including immune checkpoint inhibitors, adoptive cell therapies, and cytokine-based therapies. Understanding the metabolic requirements and dysregulations in immune cells can help identify patient subgroups more likely to respond to specific immunotherapies. Furthermore, modulating immune cell metabolism through targeted interventions, such as metabolic inhibitors or activators, can enhance the effectiveness of immunotherapies and overcome resistance mechanisms.

The precision immunotherapy can benefit from the principles of immunometabolism. By characterising the metabolic phenotype of patients’ immune cells, clinicians can identify metabolic vulnerabilities that can be targeted for therapeutic purposes. This personalised approach can improve treatment outcomes, reduce adverse effects, and enhance patient satisfaction. Moreover, integrating metabolic profiling with other omics data, such as genomics and proteomics, can enable a comprehensive understanding of the molecular landscape driving immune-related disorders and facilitate the development of targeted therapies.

Advancements in epigenetic editing tools and single-cell epigenomic techniques have revolutionised the field of epigenetics and opened up new avenues for understanding the complex mechanisms underlying gene regulation and cellular identity^59^. These cutting-edge technologies offer unprecedented resolution and precision, enabling researchers to delve deeper into the epigenomic landscape and unravel the intricacies of epigenetic modifications at a single-cell level.

One of the remarkable developments in this field is the emergence of epigenetic editing tools, such as CRISPR-based systems, that allow for targeted modifications of specific epigenetic marks^72^. With high specificity and efficiency, these tools enable researchers to manipulate and modulate epigenetic marks, such as DNA methylation and histone modifications. By selectively altering epigenetic marks at specific genomic loci, researchers can investigate their functional roles in gene regulation, cellular differentiation, and disease development^72^. Epigenetic editing tools hold great promise for both basic research and potential therapeutic applications, as they can modify epigenetic states and potentially correct aberrant epigenetic patterns associated with diseases.

In parallel, single-cell epigenomic techniques have emerged as powerful tools for dissecting the heterogeneity and dynamics of epigenetic landscapes within individual cells^82^. Traditional bulk epigenomic approaches provide averaged information from a population of cells, masking cell-to-cell variations^83^. Single-cell epigenomic techniques, such as single-cell bisulfite sequencing, single-cell ATAC-seq, and single-cell ChIP-seq, enable the characterisation of epigenetic marks and chromatin accessibility at a single-cell resolution^83^. These techniques allow researchers to uncover epigenetic states and regulatory elements that drive cellular diversity, cellular transitions, and disease progression. Moreover, single-cell epigenomic approaches can shed light on epigenetic heterogeneity within cell populations, providing insights into cellular plasticity and the influence of epigenetic regulation on cell fate decisions.

The integration of epigenetic editing tools and single-cell epigenomic techniques presents an exciting opportunity to address fundamental questions in epigenetics and advance our understanding of gene regulation and cellular dynamics. By combining the ability to edit specific epigenetic marks with the ability to analyse epigenetic landscapes at a single-cell level, researchers can gain a comprehensive view of the cause-and-effect relationships between epigenetic modifications and cellular phenotypes. This integrative approach has the potential to uncover novel epigenetic mechanisms, identify key regulators of epigenetic states, and provide insights into the epigenetic basis of disease.

Furthermore, these advancements have implications for precision medicine and therapeutic interventions. The ability to precisely manipulate epigenetic marks and investigate their functional consequences in individual cells can pave the way for developing targeted epigenetic therapies. By selectively modifying epigenetic states associated with disease, it may be possible to reverse or alleviate disease-associated epigenetic dysregulation.

CAR-T-cell therapy has emerged as a highly promising immunotherapeutic strategy that harnesses the immune system’s power to combat cancer^84^. This revolutionary therapy involves genetically modifying a patient’s T cells to express chimeric antigen receptors (CARs) specific to tumour-associated antigens^84^. The engineered CAR-T cells exhibit enhanced tumour recognition and destruction capabilities, leading to remarkable clinical responses in patients with haematological malignancies^85^. Recent studies have shed light on the critical role of immunometabolism in CAR-T-cell therapy^85,86^. CAR-T cells’ activation, proliferation, and effector functions are tightly linked to metabolic reprogramming, ensuring their optimal anti-tumour activity. Metabolic pathways such as glycolysis, oxidative phosphorylation, fatty acid metabolism, and amino acid metabolism play vital roles in CAR-T-cell function and persistence within the tumour microenvironment^87^. Furthermore, the metabolic characteristics of the tumour microenvironment profoundly influence CAR-T-cell responses. Tumour cells create a hostile metabolic landscape characterised by nutrient deprivation, hypoxia, and immunosuppressive metabolites^88^. Understanding and manipulating these metabolic interactions hold great potential for enhancing CAR-T-cell therapy efficacy and overcoming obstacles posed by the tumour microenvironment.

## Conclusion

This review has elucidated the growing significance of metabolism in modulating immune cell function. By exploring mechanisms and implications, we have uncovered the intricate relationship between intracellular metabolism and the functionalities of immune cells. Immunometabolism, an emerging field, has unveiled the dynamic changes in metabolic processes that accompany immune cell activation and govern their functions. The reprogramming of cellular metabolism plays a pivotal role in enabling immune cells to adapt and respond to diverse physiological and pathological contexts. We have provided a comprehensive overview of this topic through a synthesis of various study types, including experimental, review, and clinical investigations. Our analysis has underscored the interplay between metabolic pathways and immune cell activities, underscoring their critical roles in disease pathogenesis and potential treatment strategies. Notably, metabolic dysregulation has been implicated in immune-related disorders, offering promising avenues for targeted therapeutic interventions. A deeper understanding of the metabolic foundations of immune cell function holds considerable potential for developing novel approaches to modulate immune responses and restore immune homoeostasis. While this review has contributed to our current understanding of immunometabolism, further research is warranted to unravel the intricacies of metabolic processes and their precise effects on immune cell function. Future studies focusing on innovative therapeutic strategies and metabolic targets have the potential to revolutionise the field and pave the way for transformative approaches in immune-mediated diseases.

## Ethical approval

None.

## Consent

None.

## Sources of funding

None.

## Author contribution

G.O. and N.A.: conceptualization. Writing: all authors.

## Conflicts of interest disclosure

All authors declare no conflict of interest.

## Research registration unique identifying number (UIN)

None.

## Guarantor

Nicholas Aderinto.

## Data availability statement

Data sharing is not applicable to this article.

## Provenance and peer review

Not commissioned, externally peer-reviewed.
